# Diagnostic Performance of [^68^Ga]Ga-FAPI PET/CT for Differentiating Histologically Confirmed Kidney Fibrosis Severity in Advanced Chronic Kidney Disease

**DOI:** 10.3390/diagnostics16172706

**Published:** 2026-08-25

**Authors:** Constantin Aschauer, Johannes Kläger, Dragan Copic, Alexander Kainz, Adelina Göllner, Markus Kieler, Lukas Kenner, Michaela Schlederer, Isaia Kássimo da Costa, Maja Nackenhorst, Stefan Schmitl, Sazan Rasul, Marcus Hacker, Oana Cristina Kulterer

**Affiliations:** 1 Division of Nephrology and Dialysis, Department of Medicine III, Medical University of Vienna, 1090 Vienna, Austria; constantin.aschauer@meduniwien.ac.at (C.A.); dragan.copic@meduniwien.ac.at (D.C.); alexander.kainz@meduniwien.ac.at (A.K.); 2 Department of Pathology, Medical University of Vienna, 1090 Vienna, Austria; johannes.klaeger@meduniwien.ac.at (J.K.); lukas.kenner@meduniwien.ac.at (L.K.); michaela.schlederer@meduniwien.ac.at (M.S.); isaia.kassimodacosta@meduniwien.ac.at (I.K.d.C.); maja.nackenhorst@meduniwien.ac.at (M.N.); 3 Division of Nuclear Medicine, Department of Biomedical Imaging and Image-Guided Therapy, Medical University of Vienna, 1090 Vienna, Austria; adelina.goellner@meduniwien.ac.at (A.G.); stefan.schmitl@meduniwien.ac.at (S.S.); sazan.rasul@meduniwien.ac.at (S.R.); marcus.hacker@meduniwien.ac.at (M.H.)

**Keywords:** chronic kidney disease, imaging, fibrosis, fibroblast activation protein, PET/CT

## Abstract

**Background**: Interstitial fibrosis is a hallmark of chronic kidney disease (CKD) progression; however, its assessment currently relies on kidney biopsy, which is invasive and prone to sampling error. Fibroblast activation protein inhibitor (FAPI) positron emission tomography/computed tomography (PET/CT) targets activated fibroblasts and may enable non-invasive assessment of renal fibrosis. We investigated whether renal parenchymal [^68^Ga]Ga-FAPI uptake reflects histological fibrosis and evaluated its performance in highly fibrotic kidneys. **Methods**: In this prospective single-center study, 23 patients undergoing clinically indicated kidney biopsy underwent [^68^Ga]Ga-FAPI PET/CT imaging. Standardized uptake values (SUV) of the kidneys were matched to histological fibrosis assessments as the H-score (0–300), the fibrotic area (%) and intensity grade (0–III). Associations were calculated by Spearman rank correlation and diagnostic performance for advanced fibrosis was evaluated by ROC analysis with leave-one-out cross-validation. **Results**: SUVmean correlated with H-score (ρ = 0.71), fibrotic area (ρ = 0.79) and intensity grade (ρ = 0.74; all *p* < 0.001), whereas SUVmax (ρ = 0.17) and SUVpeak (ρ = 0.29) did not. For advanced fibrosis (intensity grade III), SUVmean discriminated with an AUC of 0.89 (95% CI 0.67–1.00). A Youden-derived cut-off of ≥2.83 yielded cross-validated sensitivity/specificity of 92%/82%. **Conclusions**: Renal parenchymal FAPI expression, represented by SUVmean, correlates with the histological severity of interstitial fibrosis, supporting [^68^Ga]Ga-FAPI PET/CT as a non-invasive tool to potentially monitor renal fibrosis over time. FAPI expression is closely linked to renal function, and subsequent larger studies are needed to validate these findings and the proposed exploratory cut-off value.

## 1. Introduction

The incidence of chronic kidney disease (CKD) is increasing in the modern world, primarily due to the increasing prevalence of diabetes, hypertension, and obesity making CKD a significant burden to the healthcare system [1,2]. Furthermore, autoimmune diseases such as IgA glomerulonephritis or vasculitis also significantly contribute to the prevalence of CKD [3,4,5,6]. 

Modern therapeutic approaches such as novel targeted immune therapies improve the outcome by reducing disease progression and by sustaining organ function but also by reducing fibrosis [7,8,9,10]. These novel therapies are associated with substantial costs and, particularly in the case of immunosuppressive agents, require careful patient selection. As CKD may continue to progress despite treatment, the potential therapeutic benefits must outweigh the risks of adverse events, including infections and malignancies [11,12,13,14,15].

Histologically, disease progression is accompanied by fibrotic tissue remodeling, leading to GFR reduction [4,7,16]. The decision to start or continue an immunosuppressive therapy can often depend on the already present amount of chronic damage in the kidney, often not reliably reflected by serum creatinine, and can ultimately only be evaluated by histological analyses obtained through kidney biopsy. This invasive procedure is often contraindicated or delayed for several reasons, such as essential platelet inhibition or anticoagulation. Furthermore, post-procedural hospitalizations are necessary as complications, especially significant bleeding might occur [17,18,19]. However, histological evaluation also has its caveats. Interpretation can be limited by insufficient tissue collection or sampling errors as fibrosis can be unequally distributed in kidney diseases [20,21,22,23]. Moreover, patients with ongoing diseases requiring continuous therapy are subjected to numerous biopsies over the course of their disease to assess disease activity, monitor progression and evaluate the extent of chronic kidney damage.

Advances in imaging techniques to non-invasively evaluate kidney fibrosis such as functional MRI and elastin imaging or shear wave elastography have given promising results but have not yet been introduced to daily clinical use [24,25,26]. In the past few years, with the advent of higher spatial resolution and the increased availability of tracers, PET/CT imaging has emerged as a useful tool in the diagnosis of infections or acute rejection episodes after kidney transplantation [26,27]. Newer studies have evaluated upcoming tracers such as [^68^Ga]Ga-FAPI in different chronic and fibrotic diseases of other organs and have proven to reliably assess the degree of fibrosis in diseases such as Crohn’s disease and ILDs [28,29,30]. Nevertheless, as radioactive tracers are mostly renally excreted, the visualization of the kidney is often difficult and data on most tracers for the evaluation of renal pathology are scarce.

A recent retrospective study showed that FAPI PET uptake correlates with eGFR [31]. Further studies in patients undergoing kidney biopsies showed that [^68^Ga]Ga-FAPI PET/CT is a potential tool in the visualization of renal fibrosis, but data for patients with advanced fibrosis are missing [32,33,34]. 

Therefore, we conducted a prospective study in patients undergoing kidney biopsy to evaluate the association between histologically quantified renal fibrosis and FAPI expression on PET/CT. 

## 2. Materials and Methods

### 2.1. Patient Recruitment

Twenty-three patients were prospectively enrolled after undergoing clinically indicated kidney biopsy at our center. The underlying kidney biopsies were performed between November 2024 and April 2026. All biopsies were performed exclusively for routine clinical indications, representing most commonly the diagnostic work-up of proteinuria or glomerular hematuria and unexplained decline of renal function but also evaluation of continuing immunosuppressive therapies (patients 4, 6 and 11; Supplemental Table S1). Biopsies were not performed as part of the research protocol. Only patients who underwent kidney biopsies were enrolled in this clinical study.

Patients were included according to the degree of interstitial fibrosis found in the histologic work-up which spanned the whole range of no/nearly absent interstitial fibrosis to diffuse and strong interstitial fibrosis (Supplemental Table S2). Patient characteristics and comedications are shown in Table 1 and Supplemental Table S3, respectively. All patients gave written informed consent prior to study interventions.

### 2.2. Radiopharmaceuticals

For imaging the radiotracer [^68^Ga]Ga-DOTA.SA.FAPI, a squaric-acid, quinolone-based molecule with in vitro FAP inhibiting activity that has demonstrated high diagnostic performance and favorable tracer kinetics, was used. The synthesis procedure of the radiotracer was carried out at the Department of Biomedical Imaging and Image-guided Therapy, Division of Nuclear Medicine as described previously [30].

### 2.3. PET Imaging

PET/CT imaging was performed at least 34 days and at most 410 days after kidney biopsy to avoid unspecific tracer uptake following the procedure.

Patients underwent [^68^Ga]GaFAPI PET/CT imaging at the Department of Biomedical Imaging and Image-guided Therapy, Division of Nuclear Medicine of the Medical University of Vienna. All PET/CT scans were acquired with a Siemens Biograph 128 Vision Quadra Edge (Siemens Healthineers, Knoxville, TN, USA). In every examination, whole body scans from the skull to the mid-thigh were obtained 45–60 min after the tracer injection. Each patient received approximately 100 MBq [^68^Ga]Ga-DOTA.SA.FAPI. No standardized hydration protocol or routine pre-scan diuretic administration was used and no correction for hydration status was performed. The CT acquisition protocols for the PET scans followed the standardized institutional imaging protocol and were consistent with the routine clinical practice at our institution: First, a low-dose CT scan was acquired between 80 and 120 kV and 30–40 mAs. A CT matrix of 512 × 512 and slice thickness of 2 mm were used in all scans. The examinations were performed with a duration of 4.5 min in one position with a field of view (FOV) of 106 cm employing an iterative reconstruction technique that utilized a point-spread-function-based algorithm. Subsequent to this, corrections for scatter and attenuation were applied based on the CT scan (PET matrix size: 440 × 440).

Image analysis was conducted using the Hermes Affinity Software (Version 2.18; Hermes Medical Solutions, Stockholm, Sweden). PET image intensities (Bq/mL) were transformed into standardized uptake values (SUV) normalized to body weight. SUVmean, SUVmax (maximum) and SUVpeak values for the organs (kidney and liver) were obtained in a consensus image interpretation by two experienced nuclear medicine specialists, who were blinded to the histological scoring and clinical data during image analysis. The kidneys were manually delineated in order to avoid spill-over from the renal pelvis and collecting system. The liver was automatically delineated and revised by the nuclear medicine physicians. 

Any focal uptake higher than the surrounding background within the kidney tissue was considered pathologic. The nuclear medicine specialists were unaware of the histological scoring and clinical data at the time of analysis.

### 2.4. Histologic Work-Up

Histological sections obtained during routine diagnostic work-up were retrieved from the archive. This routine work-up of biopsies consisted of serial sections with at least two acid fuchsin orange stainings on which detailed evaluation of interstitial fibrosis was performed. Fibrosis was defined as cortical areas with tubules not lying back-to-back. Fibrosis was quantified using three approaches (Supplemental Figures S1 and S2): (1) the percentage of any fibrotic area of the cortical part of a biopsy core by visual assessment (in 10% increments), (2) an ordinal interstitial fibrosis intensity grade by visual assessment of distension of tubules and staining intensity (0 = absent/nearly absent, I = mild, II = moderate, III = severe)—the most abundant grade was chosen, and (3) an H-score (0–300) derived from fibrotic area percentages and corresponding severity grades (0–3). The pathologists assessing interstitial fibrosis were unaware of the [^68^Ga]Ga-FAPI PET/CT results. To assess the reproducibility of the histological reference standard scored by renal pathologists, an interobserver agreement analysis was performed. It was quantified by two-way intra-class correlation coefficients (single measure, absolute agreement) for the H-score and fibrotic area, by weighted kappa for the intensity grade, and by Cohen’s kappa for the classification of advanced fibrosis, with percentile bootstrap 95% confidence intervals (10,000 resamples) and can be found in the Supplemental Material (Supplemental Table S4). 

### 2.5. Statistical Analysis

The Spearman rank correlation coefficient was used to quantify the associations between the renal PET parameters (SUVmean, SUVmax and SUVpeak) and the histological measures of fibrosis (H-score, fibrotic area and intensity grade). Normality of the variables was assessed with the Shapiro–Wilk test, and due to non-normal distribution, a non-parametric coefficient was chosen [33,35]. The standardized uptake values of the biopsied kidney were matched to the histology and 95% confidence intervals were derived from the Fisher z-transformation using the Bonett–Wright variance for rank correlations [36,37]. 

Continuous data are presented as mean ± standard deviation or median with interquartile range (IQR), and categorical data as counts and percentages. The same Spearman rank correlation coefficient was used to relate SUVmean to renal function (serum creatinine and eGFR) and to the uptake of the contralateral kidney. Partial Spearman rank correlations, adjusting for serum creatinine and, separately, for eGFR, were additionally computed to assess the association between SUVmean and the histological fibrosis measures independent of renal function. Multiplicity across the PET-versus-histology comparisons was controlled with the Benjamini–Hochberg false-discovery rate, and differences in uptake between fibrosis groups were tested with the Kruskal–Wallis and Mann–Whitney U tests [32]. Baseline clinical characteristics and medication were compared between patients with and without advanced fibrosis using the same tests (Supplemental Table S5). The diagnostic performance of SUVmean for advanced fibrosis (pre-specified as an H-score ≥ 200 and, separately, as an intensity grade of III) was evaluated by receiver-operating-characteristic (ROC) analysis, and a candidate cut-off was identified with the Youden index. To estimate how this cut-off would perform in new patients, sensitivity and specificity were additionally obtained by leave-one-out cross-validation and are reported as internally validated, out-of-sample estimates. 95% confidence intervals for the AUC were obtained by the percentile bootstrap with 5000 case resamples (patients resampled with replacement; resamples containing a single outcome class were discarded), using the NumPy PCG64 generator with a fixed seed.

The association of SUVmean with each binary outcome was additionally modeled with Firth penalized logistic regression, which is robust in small samples and in the presence of separation. A two-sided *p*-value < 0.05 was considered statistically significant. All analyses were performed in Python 3.12 (NumPy 2.4, SciPy 1.17, scikit-learn 1.8) and Claude (Anthropic; Opus 4.8) was used to assist with optimizing graphical illustrations and with statistical analysis. 

## 3. Results

### 3.1. Patient Characteristics

Initially, twenty-seven patients were included in the study and four patients withdrew consent. Finally, the study cohort included fifteen men and eight women with a mean age of 46.9 ± 14.8 years. Renal function at imaging ranged widely, with a median serum creatinine of 1.8 mg/dL (IQR 1.2–3.1) and a median eGFR of 48.3 mL/min/1.73 m^2^ (20.8–72.5). Eighteen patients presented with proteinuria with a median protein–creatinine ratio of 920 mg/g (404–1900), a median albumin-creatinine ratio of 663 mg/g (204–1505), and nine of the 23 patients presented with hematuria. The biopsy was obtained from the left kidney in twenty patients and the right kidney in three patients. Patients’ characteristics, comedications, histologic findings and differences in baseline characteristics are shown in Table 1 and Supplemental Tables S1–S3, S5 and S6. 

### 3.2. FAPI Expression Is Increased in Patients with Renal Fibrosis 

**Figure 1:**
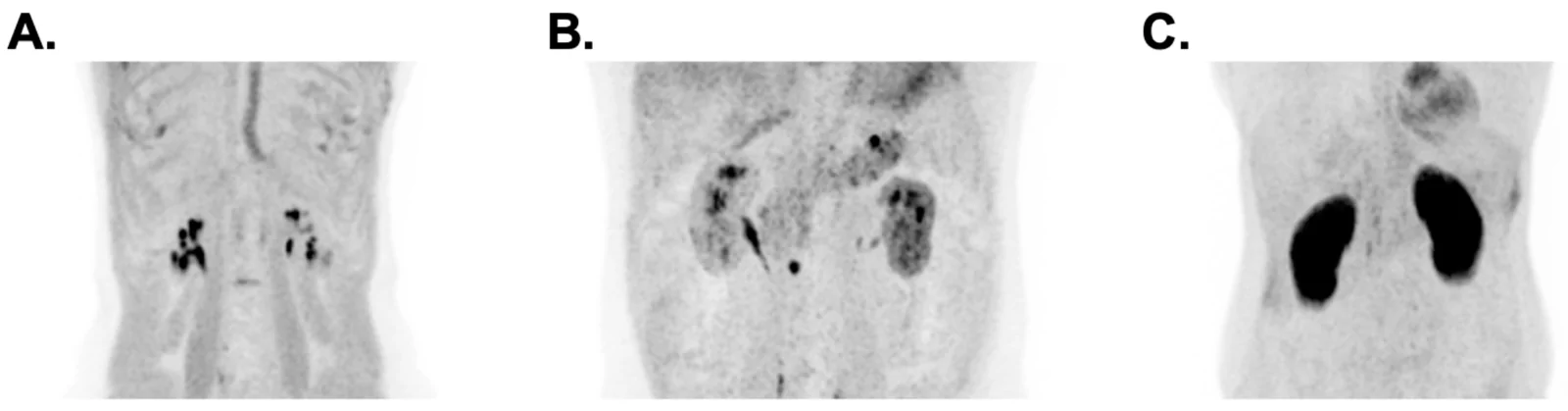
Renal FAPI expression is increased in patients with interstitial fibrosis. Representative [^68^Ga]Ga-FAPI PET images showing renal FAPI expression in patients with no interstitial fibrosis (**A**), moderate (**B**) and severe interstitial fibrosis (**C**). Physiologic FAP expression in the esophagus, uterus, liver and heart.

Renal parenchymal FAPI uptake was present in all twenty-three patients and ranged widely, from low uptake in kidneys without fibrosis to intense uptake in advanced fibrosis (Figure 1A–C). PET-derived metrics and histological scores for interstitial fibrosis intensity grade are shown in Supplemental Table S2. 

**Figure 2:**
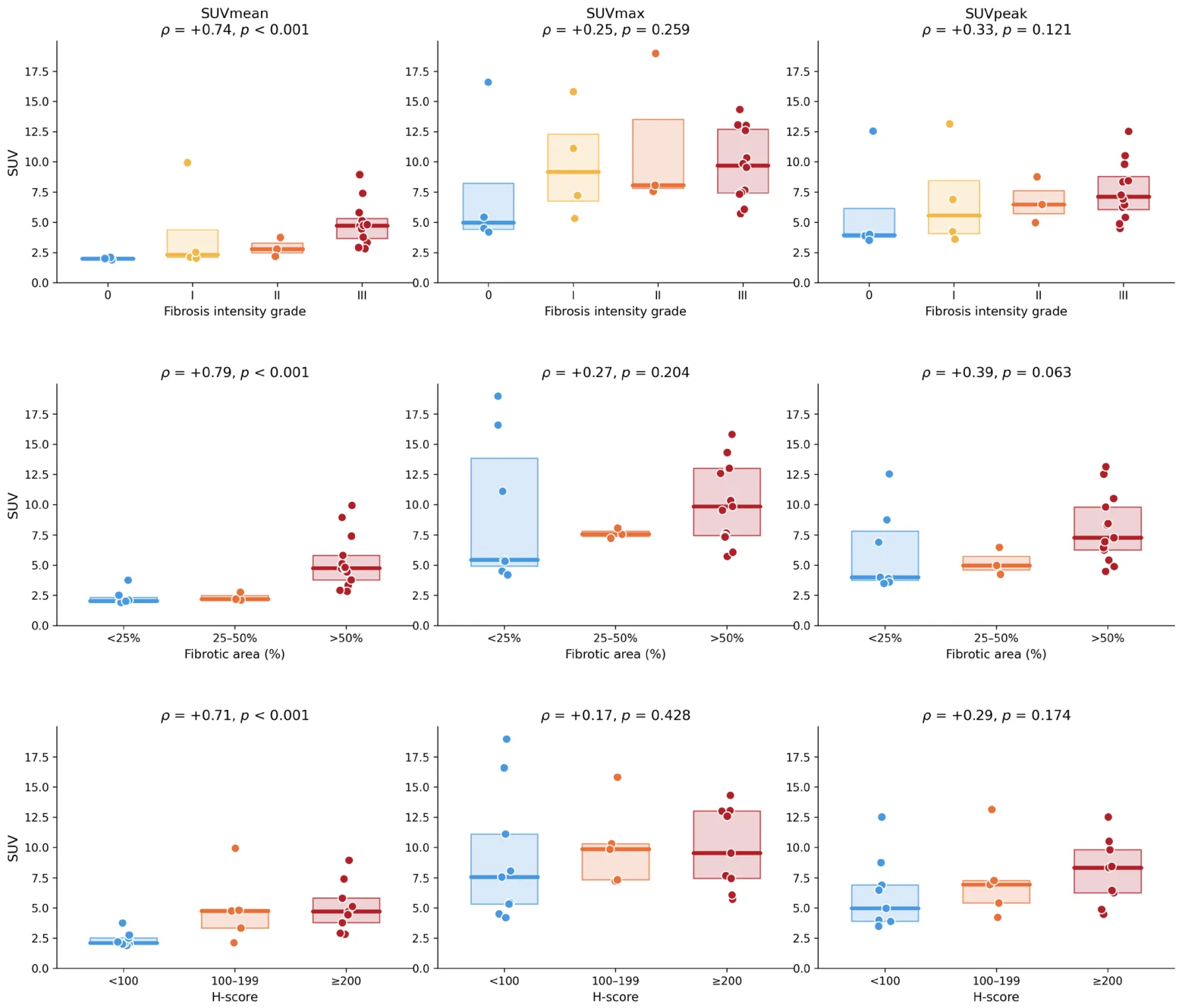
SUVmean, SUVmax, SUVpeak and histologic assessment of included patients. SUVmean increases with the degree of fibrosis found in the kidney biopsy, evaluated by fibrosis intensity grade, H-score and fibrotic area. Shown are the values of the biopsied kidney and patients are ordered on behalf of their fibrotic intensity grade, fibrotic area and H-score.

Median [IQR] SUVmean was 1.98 [1.95–2.02] in grade 0 (n = 4), 2.31 [2.09–4.38] in grade I (n = 4), 2.77 [2.48–3.26] in grade II (n = 3) and 4.72 [3.66–5.29] in grade III (n = 12), whereas SUVmax was 4.96 [4.42–8.22] in grade 0, 9.18 [6.76–12.29] in grade I, 8.07 [7.81–13.53] in grade II and 9.70 [7.42–12.70] in grade III. SUVpeak was 3.94 [3.79–6.13], 5.56 [4.07–8.46], 6.48 [5.72–7.62] and 7.11 [6.04–8.79] for grades 0/I/II/III, respectively. The H-score increased across grades, from 10 [10–10] (grade 0) to 210 [197.5–275] (grade III), and the fibrotic area from 0% [0–0]% to 85% [70–100]% (Figure 2).

SUVmean was significantly higher in patients with interstitial fibrosis (grade I–III) than in those without signs of fibrosis (3.77 [2.80–4.97] vs. 1.98 [1.95–2.02]; Mann–Whitney *p* < 0.001), whereas SUVmax (9.54 [7.39–12.80] vs. 4.96 [4.42–8.22]; *p* = 0.14) and SUVpeak (6.89 [5.20–8.61] vs. 3.94 [3.79–6.13]; *p* = 0.16) did not differ significantly between the two groups. The maximum- and peak-based metrics were also elevated in fibrosis-free kidneys and did not rise consistently with grade (Supplemental Figure S3). Consistently, when stratified by H-score, SUVmean increased from minimal (<100; median 2.10) through moderate (100–199; median 4.74) to advanced (≥200; median 4.70) fibrosis (Kruskal–Wallis *p* = 0.002). The same graded increase was evident when patients were stratified by fibrotic area (mild < 25%, median SUVmean 2.02; moderate 25–50%, 2.18; severe >50%, 4.74) or by intensity grade (grades 0/I/II/III, medians 1.98/2.31/2.77/4.72). Taken together these data support the notion that FAP expression as depicted by SUVmean is a robust marker for the in vivo detection and quantification of interstitial fibrosis.

### 3.3. Renal FAP Expression Is Associated with the Severity of Interstitial Fibrosis 

**Figure 3:**
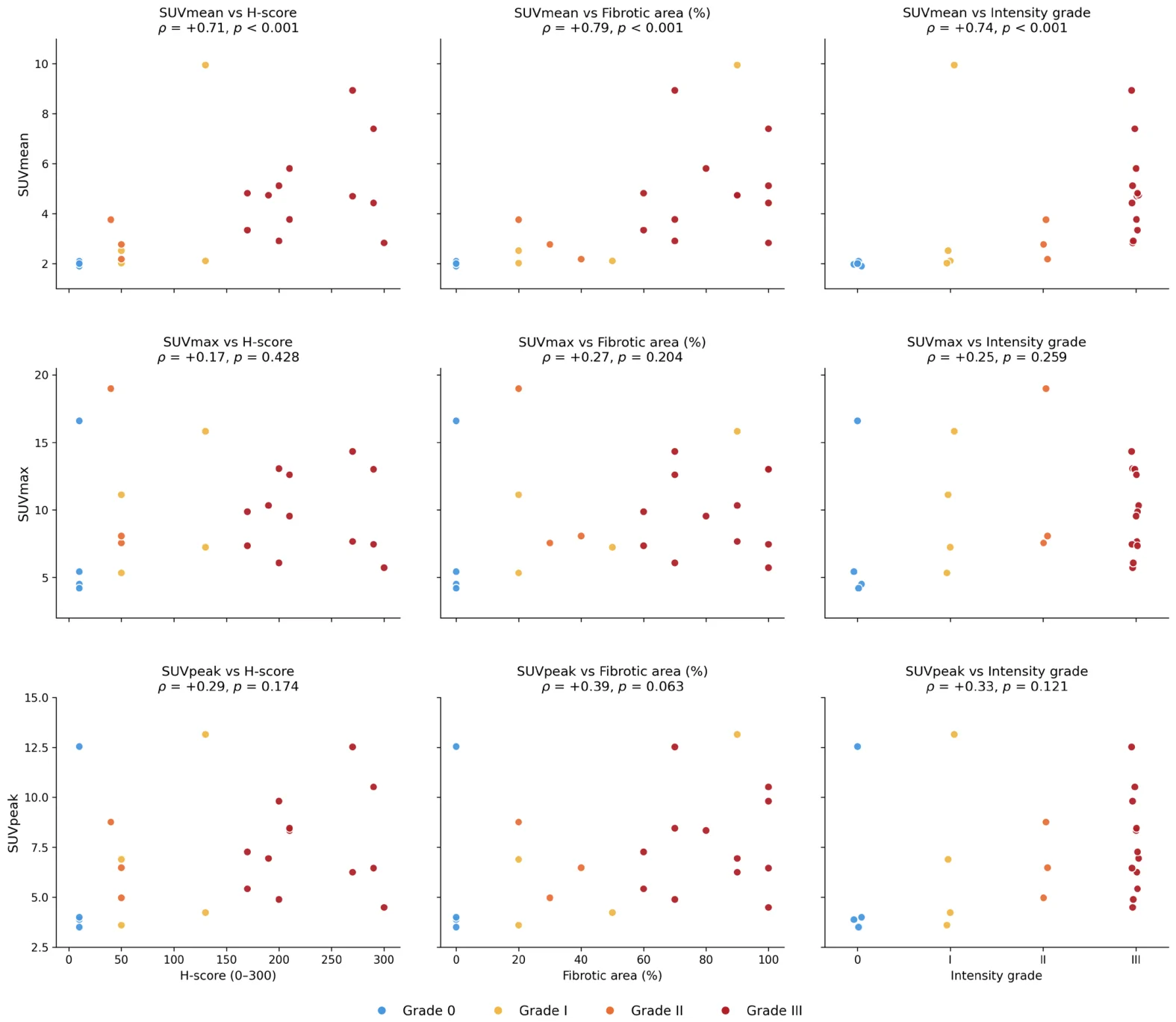
Renal FAPI uptake vs. histologic fibrosis assessment. SUVmean shows significant correlation to all three histologic parameters (**top row**); SUVmax and SUVpeak do not (**middle**, and **lowest rows**, respectively).

Renal parenchymal SUVmean of the biopsied kidney correlated positively with all three histological parameters of fibrosis: the H-score (Spearman ρ = 0.71, *p* < 0.001), the fibrotic area (ρ = 0.79, *p* < 0.001) and the interstitial-fibrosis intensity grade (ρ = 0.74, *p* < 0.001) (Figure 3). 

In contrast, neither SUVmax (ρ = 0.17, *p* = 0.43) nor SUVpeak (ρ = 0.29, *p* = 0.17) correlated significantly with the H-score or the interstitial fibrosis grade, indicating that the mean rather than the maximal parenchymal uptake reflects diffuse fibrosis (Table 2). This difference was mainly driven by an outlier (patient 12) whose SUVmax was 16.6 despite grade 0 fibrosis. Among the thirteen histological entities included, tracer uptake appeared to reflect the degree of histological fibrosis rather than the underlying disease group, with a mean SUVmean of 9.45 in IC-MPGN, 5.28 in diabetic nephropathy, 4.67 in FSGS, 3.49 in subacute tubular injury and 3.34 in IgA nephropathy, versus 2.00 in thin basement membrane disease (n = 2–3 per group). Of note, SUVmean was also associated with renal function, correlating positively with serum creatinine (ρ = 0.91, *p* < 0.001) and inversely with eGFR (ρ = −0.90, *p* < 0.001). Consequently, after adjustment for serum creatinine, the partial correlation of SUVmean with the H-score was (ρ = −0.13, *p* = 0.56), and after adjustment for eGFR (ρ = −0.20, *p* = 0.37) and partial correlations with the fibrotic area and the intensity grade were likewise not significant (all *p* ≥ 0.19).

### 3.4. Evaluation of SUVmean Cut-Off Values for High-Grade Fibrosis

To explore whether a single SUVmean value could distinguish kidneys with advanced fibrosis, candidate cut-offs were derived and internally validated. SUVmean was evaluated against severity strata and against dichotomous definitions of advanced fibrosis for all three measures. SUVmean differed significantly across severity strata for the H-score (minimal < 100/moderate 100–199/advanced ≥200; Kruskal–Wallis H = 12.55, *p* = 0.002), the fibrotic area (<25%/25–50%/>50%; H = 15.00, *p* < 0.001) and the intensity grade (0–III; H = 12.77, *p* = 0.005). A common exploratory SUVmean threshold of ≥2.83, identified by the Youden index, best separated advanced from non-advanced fibrosis under every definition. SUVmean reached an AUC of 0.98 (95% CI 0.90–1.00; leave-one-out cross-validated sensitivity/specificity 92%/90%) for a fibrotic area > 50%, 0.89 (0.67–1.00; 92%/82%) for intensity grade III, and 0.82 (0.61–0.97; 78%/64%) for an H-score ≥ 200 (Table 3). These estimates were consistent on bootstrap resampling (5000 case resamples, fixed seed), which yielded comparable AUCs of 0.977 (95% CI 0.908–1.000) for the fibrotic area, 0.887 (0.669–1.000) for intensity grade III and 0.815 (0.607–0.970) for the H-score (Supplemental Table S7 and Supplemental Figure S4).

Using Firth penalized logistic regression, the odds of grade III fibrosis increased with SUVmean (odds ratio 1.53 per unit, 95% CI 1.00–3.03). Discrimination was thus consistent across all three histological references, being highest for the fibrotic area and lowest for the H-score. 

## 4. Discussion

In this prospective exploratory study, we investigated the diagnostic value of [^68^Ga]Ga-FAPI PET/CT to predict interstitial fibrosis in clinically and histologically proven kidney disease. We were able to show that the FAP expression correlated with the histological severity of interstitial fibrosis, measured by the fibrotic area, the ordinal intensity grade or H-score. 

Until now [^68^Ga]Ga-FAPI PET/CT has been assessed in only a few renal cohorts with concomitant histologic analysis of the kidneys. Zhou et al. showed a stepwise increase in tracer uptake across mild, moderate and severe renal fibrosis, and Wang et al. reported positive correlations of both SUVmax and SUVmean with interstitial fibrosis and tubular atrophy in IgA nephropathy [32,33]. The same group subsequently extended this to a mixed group of renal pathologies, again with both SUVmax and SUVmean correlating with fibrosis [34]. Conen et al. related uptake only to the glomerular filtration rate, due to the lack of histology, and a recent lupus-nephritis study quantified uptake as a target-to-background ratio rather than as SUVmean or SUVmax, hampering direct comparisons [35,38].

Across these cohorts, the degree of fibrosis was comparatively low and renal function comparatively preserved. Zhou et al. included only three patients with high-grade fibrosis, the IgA-nephropathy patients of Wang et al. had a maximal tubular-atrophy score of 1 (mean eGFR 73 mL/min/1.73 m^2^), and the mixed cohort of Wang et al. comprised predominantly early-stage disease (mean eGFR 65 mL/min/1.73 m^2^). In contrast, our cohort represents a broader range of fibrosis including advanced fibrosis and impaired function with eight of twenty-three patients exceeding 70% fibrotic area and a median eGFR of 48.3 mL/min/1.73 m^2^.

Interestingly, in our cohort, only SUVmean showed a significant correlation with histological assessment of fibrosis, whereas neither SUVmax nor SUVpeak did, in contrast to previous studies. One possible explanation is that fibrosis is typically a diffuse and spatially heterogeneous process rather than a focal lesion. SUVmean reflects the average tracer uptake across the entire segmented tissue, thereby providing a more comprehensive measure of the overall fibrotic burden. In contrast, SUVmax is based on a single voxel and is therefore more susceptible to image noise, statistical fluctuations, and localized hotspots that may not accurately represent the extent of fibrosis. Similarly, although SUVpeak is less sensitive to noise than SUVmax, it still emphasizes regions of highest uptake rather than the global tissue characteristics. Another possible explanation could be the patient characteristics.

In other cohorts, the patients analyzed were dominated by IgA nephropathy which can present focal and segmental lesions, potentially leading to focal high-uptake areas, in contrast to the mixed etiology in our cohort, which reflects a largely diffuse fibrotic burden, leading to SUVmean being more adequate [38,39,40]. Also, the ^68^Ga-labeled tracer used in this cohort has a longer positron range, which lowers spatial resolution relative to the ^18^F-labelled FAPI tracers and can therefore lead to less detailed SUVmax measurements [41]. 

In our study, we included twenty-three patients with thirteen different renal fibrotic diseases. Our data suggest that FAPI uptake may be more closely related to the degree of fibrotic remodeling than to the underlying renal disease itself. Nevertheless, the underlying etiology may potentially influence the pattern of tracer uptake as diseases with patchy or segmental fibrosis, as IgA nephropathy, polyomavirus nephropathy, or focal scarring after pyelonephritis or renal embolism may result in a lower whole-kidney SUVmean at a given histological severity than the diffuse fibrosis typical of hypertensive or diabetic nephropathy. Of note is the fact that SUVmean was also associated with renal function, correlating positively with serum creatinine and inversely with eGFR, which is in line with previous studies [33,34,35]. The attenuation of the SUVmean-histology fibrosis association after adjustment for renal function seen in the analysis is expected, as interstitial fibrosis is per se a principal driver of the loss of renal function. 

Interestingly, all three patients who progressed to dialysis shortly after imaging (patients 4, 6, 11) had the three highest SUVmean values in the cohort, and additionally patients 7, 17 and 20 with poor renal function could be found above the exploratory SUVmean threshold. Also of interest, patient 6 presented histologically with a rather low H-score of 130 but advanced to dialysis and patient 17, with end-stage 100% fibrosis, showed rather low SUVmax values but reached the SUVmean threshold. Nevertheless, this threshold is exploratory and cannot be used in clinical decision making before larger external validation. 

Major limitations of the study were the relatively small sample size, the lack of an a priori sample size calculation leading to a lack of statistical power and the single center design at a tertiary referral center, potentially leading to selection bias. However, given the exploratory nature of this study and the limited available evidence on the use of [^68^Ga]Ga-FAPI PET/CT for the assessment of high-grade renal fibrosis, the cohort size was considered appropriate for this initial investigation and hypothesis-generating analysis. 

To our knowledge, this cohort represents one of the largest collective published, with the most advanced kidney impairment and the most severe fibrosis. This reflects cases where clinical decisions regarding indication for therapy continuation or discontinuation are relevant. Another limitation of this study was the extended interval between biopsy and [^68^Ga]Ga-FAPI PET/CT scan. Therefore, changes in disease activity or initiation of treatment during this interval may have influenced the degree of fibrosis and tracer uptake in individual patients. However, renal fibrosis generally represents a slowly evolving pathological process, making substantial changes in fibrosis burden over relatively short periods less likely. Furthermore, we did not observe significant changes in serum creatinine or eGFR between biopsy and imaging in this cohort. 

Another limitation of the study is that there was no adjustment for diuretic use although it could influence tracer distribution. However, loop-diuretic therapy, titrated to clinical volume status, was numerically more frequent in advanced fibrosis without reaching significance (Supplemental Table S5). Data on the influence of diuretics on renal FAPI uptake are lacking, although for [^68^Ga]Ga-PSMA PET/CT furosemide markedly reduced urinary bladder activity while renal parenchymal SUV did not change significantly [42].

A strength of this study is that histology was quantified using different scorings, reflecting ordinal but also continuous grading, and potential heterogeneity of fibrotic intensity, and our negative controls were biopsy-proven, which was not the case in the only larger cohort published recently. However, future prospective, multicentric longitudinal studies are needed to further validate the role of FAPI imaging in renal fibrosis. Another important future perspective is the integration of [^68^Ga]Ga-FAPI with complementary imaging modalities such as multiparametric renal MRI and ultrasound elastography and with emerging serum and urinary biomarkers within a multimodality imaging framework, which could potentially allow simultaneous assessment of molecular fibroblast activation, tissue composition, and structural alterations. Such multiparametric approaches may ultimately improve non-invasive characterization of renal fibrosis and help distinguish active, potentially modifiable fibrogenesis from established irreversible fibrosis. Yet at present the comprehensive histopathological assessment of the kidney remains indispensable [43].

In conclusion, in our study conducted in a heterogeneous cohort of patients with fibrotic renal diseases, we demonstrated that FAPI renal uptake correlated with the histological severity of interstitial fibrosis. Moreover, [^68^Ga]Ga-FAPI PET/CT identified patients with clinically more advanced disease, supporting the potential of whole-organ FAPI imaging to detect renal fibrosis and non-invasively capture the global fibrotic burden of the entire kidney. We therefore propose [^68^Ga]Ga-FAPI PET/CT imaging as a complementary, non-invasive tool capable of whole-organ fibrosis quantification. In the future [^68^Ga]Ga-FAPI PET/CT might be particularly relevant in clinically challenging situations such as suspected sampling error, discrepancy between biopsy and clinical course, diseases with a patchy intrarenal distribution or longitudinal fibrosis surveillance. 

## Figures and Tables

**Table 1:** Baseline demographic and clinical characteristics of the study cohort.

ID	Age (y)	Height (cm)	Weight (kg)	Sex	Days Since Bx	Bx Side	Creatinine (mg/dL)	eGFR (mL/min/1.73 m^2^)	PCR (mg/g)	ACR (mg/g)	Hematuria	Biopsy Diagnosis
1	47	165	58	F	52	Left	0.71	105	407	220	−	Thin basement disease *
2	21	158	52	F	68	Right	1.3	60	730	512	−	Unclear **
3	44	155	63	F	88	Left	4.2	13	2000	1510	−	SLE-LN III-V; not active
4	43	178	66	F	80	Left	10.3	4	11,174	8759	+	IC-MPGN
5	46	162	69	F	69	Left	0.55	114	400	217	+	Thin basement disease ***
6	57	180	93	M	148	Left	11.4	5	13,090	8139	+	IC-MPGN
7	37	150	62	F	129	Right	4.05	14	9347	6711	−	FSGS ****
8	66	175	88	M	194	Left	2.8	24	145	25	−	subacute tubular injury
9	28	164	69	F	410	Left	0.61	125	0	0	−	MC-GN
10	21	172	68	F	46	Left	0.63	129	240	90	+	Interstitial Nephritis
11	33	177	66	M	140	Left	9.6	7	9104	5752	−	FSGS ^+^
12	36	193	92	M	82	Left	0.86	115	2700	2235	−	Membranous. GN, PLA2R pos
14	63	175	80	M	60	Left	1.3	62	920	663	−	Unspecific Glomerulopathy ^++^
16	56	174	89	M	214	Right	1.3	64	90	11	−	subacute tubular damage, medullary calcium deposits, atherosclerosis
17	63	170	80	M	204	Left	2.75	25	580	190	−	subacute tubular damage ^+++^
20	75	182	96	M	172	Left	3.5	17	1800	1240	−	D-NP, HTN
21	44	168	95	M	60	Left	2.59	30	897	622	−	TMA, chronic
22	42	172	62	M	34	Left	2.8	28	713	452	−	D-NP
23	70	172	87	M	34	Left	1.15	68	1300	1000	+	Unspecific glomerulopathy; ICH neg
24	50	183	92	M	74	Left	1.48	57	1200	1000	+	Unspecific glomerular lesions
25	36	190	118	M	277	Left	1.25	77	1800	1500	+	FSGS, thin basement membrane disease? ^++++^
26	43	168	66	M	134	Left	1.77	48	185	117	+	Mesangial proliferative IgA Glomerulopathy (Oxford: M1,E0, S1, T2, C0)
27	57	177	90	M	83	Left	2.2	34	1563	1334	+	IgA-NP; no oxford score-small biopsy

Data are shown per patient at the time of imaging; Bx: biopsy; eGFR: estimated glomerular filtration rate (CKD-EPI 2021); PCR: urinary protein-to-creatinine ratio; ACR: urinary albumin-to-creatinine ratio; F: female; M: male; Hematuria: − absent, + present. Biopsy-diagnosis abbreviations: SLE-LN: systemic lupus erythematosus with lupus nephritis (ISN/RPS classes III–V); IC-MPGN: immune-complex-mediated membranoproliferative glomerulonephritis; FSGS: focal segmental glomerulosclerosis; MC-GN: minimal-change glomerulonephritis; GN: glomerulonephritis; PLA2R: phospholipase A2 receptor; D-NP: diabetic nephropathy; HTN: arterial hypertension; TMA: thrombotic microangiopathy; IgA-NP: IgA nephropathy; Oxford M/E/S/T/C, Oxford (MEST-C) classification of IgA nephropathy. Additional findings: * genetics: HBB c.93-21G>A, heterozygous, pathogenic; CUBN p.Cys2032Tyr, heterozygous, version of uncertain significance; CUBN c.10764+1G>A, heterozygous, VUS; ** high-grade focal fibrosis; 80% podocyte foot-process effacement (FPE); *** genetic testing pending; **** genetic testing pending; ^+^ genetic testing pending; ^++^ immunohistochemistry negative for immune deposits; ^+++^ hereditary transthyretin amyloidosis with cardiomyopathy (ATTRv-CM), type 2 diabetes mellitus; ^++++^ genetics negative (whole-exome sequencing, WES).

**Table 2:** Correlation analysis of renal parenchymal SUVs and histological fibrosis.

PET Parameter	Histology	ρ	*p*	95% CI	FDR q
SUVmean	H-score	0.709	<0.001	0.38 to 0.88	<0.001
SUVmean	Fibrotic area%	0.786	<0.001	0.51 to 0.92	<0.001
SUVmean	Intensity grade	0.735	<0.001	0.42 to 0.89	<0.001
SUVpeak	H-score	0.293	0.174	−0.14 to 0.64	0.261
SUVmax	H-score	0.174	0.428	−0.26 to 0.55	0.428

SUVmean correlated with all three histological measures of fibrosis, whereas SUVmax and SUVpeak did not. Each row reports the Spearman rank-correlation coefficient (ρ) between an imaging parameter (SUVmean, SUVmax or SUVpeak) and a histological measure (H-score, fibrotic area or intensity grade), with its two-sided *p*-value, 95% confidence interval and Benjamini–Hochberg false-discovery-rate-adjusted q-value. Confidence intervals were obtained from the Fisher z-transformation using the Bonett–Wright variance for rank correlations. All parameters were obtained in the biopsied kidney. The histological measures of interstitial fibrosis are the H-score (0–300), the fibrotic area (%) and the ordinal interstitial-fibrosis intensity grade (0–III). CI, confidence interval; FDR, false-discovery rate; SUV, standardized uptake value.

**Table 3:** Exploratory diagnostic performance of renal parenchymal SUVmean for the identification of advanced fibrosis.

Outcome	AUC (95% CI)	Cut-off	Estimate	Sens	Spec	PPV	NPV
H-score ≥ 200	0.817 (0.607–0.97)	≥2.83	apparent	100.0%	64.3%	64.3%	100.0%
			LOOCV	77.8%	64.3%	58.3%	81.8%
Intensity grade III	0.886 (0.669–1)	≥2.83	apparent	100.0%	81.8%	85.7%	100.0%
			LOOCV	91.7%	81.8%	84.6%	90.0%
Fibrotic area > 50%	0.977 (0.908–1)	≥2.83	apparent	100.0%	90.0%	92.9%	100.0%
			LOOCV	92.3%	90.0%	92.3%	90.0%

Advanced fibrosis was defined as an H-score ≥ 200 (9 of 23 patients) or, as an interstitial-fibrosis intensity grade of III (12 of 23 patients). Shown are the area under the ROC curve (AUC) with its bootstrap percentile 95% confidence interval (5000 resamples, fixed seed), the SUVmean cut-off identified by the Youden index, and the resulting sensitivity, specificity, and positive and negative predictive values, given both as apparent (resubstitution) values and as leave-one-out cross-validated (LOOCV) values. AUC, area under the curve; CI, confidence interval; LOOCV, leave-one-out cross-validation; NPV, negative predictive value; PPV, positive predictive value; SUV, standardized uptake value.

## Data Availability

Patient information will be stored at the main data repository of the general hospital of Vienna, following international standards of patient data security in accordance with the Austrian Data Protection Act. Biopsy samples are stored at the Department for Pathology of the General Hospital/Medical University of Vienna, where samples are labeled with a specific patients’ identifier. The decrypting code is separated strictly from the encoded records. Access to clinical and imaging data and biological samples will be limited to authorized persons. The datasets used and analyzed during the current study and the complete analysis code, including the fixed random seed and the exact bootstrap implementation used to generate all reported confidence intervals, are available from the corresponding author on reasonable request.

## Supplementary Materials

The following supporting information can be downloaded at https://www.mdpi.com/article/10.3390/diagnostics16172706/s1, Figure S1: Acid fuchsin orange-G Staining of two examples of varying amount of interstitial fibrosis; Figure S2: Acid fuchsin orange-G Staining of two examples of varying amount of interstitial fibrosis illustrating intensity scoring; Figure S3: SUVmean, SUVmax, SUVpeak assessment of included patients; Figure S4: bootstrap distribution of the AUC of renal parenchymal SUVmean for the identification of advanced interstitial fibrosis; Table S1: biopsy indication, clinical course of renal function and disease-specific therapy; Table S2: renal FAPI PET/CT uptake parameters (both kidneys) and histological fibrosis findings of the biopsied kidney; Table S3: antihypertensive, diuretic and SGLT2-inhibitor medication at the time of PET/CT; Table S4: interobserver agreement; Table S5: baseline clinical variables according to fibrosis intensity grade; Table S6: renal parenchymal SUVmean stratified by CKD stage; Table S7: bootstrap distribution statistics.

## Abbreviations

The following abbreviations are used in this manuscript:

ACE	Angiotensin-converting-enzyme
ACR	Urinary albumin-to-creatinine ratio
ARB	Angiotensin-receptor blocker
ATTRv-CM	Hereditary transthyretin amyloidosis with cardiomyopathy
AUC	Area under the curve
BID	Twice daily
Bx	Biopsy
CI	Confidence interval
CKD	Chronic kidney disease
CKD-EPI	Chronic Kidney Disease Epidemiology Collaboration (eGFR equation, 2021)
D-NP	Diabetic nephropathy
FAPI	Fibroblast activation protein inhibitor
FDR	False-discovery rate
FPE	Foot-process effacement
FSGS	Focal segmental glomerulosclerosis
eGFR	estimated Glomerular filtration rate
GN	Glomerulonephritis
HCT	Hydrochlorothiazide
HTN	Arterial hypertension
IgA-NP	IgA nephropathy
IC-MPGN	Immune-complex-mediated membranoproliferative glomerulonephritis
ILD	Interstitial lung disease
IQR	Interquartile range
ISN/RPS	International Society of Nephrology/Renal Pathology Society
LOOCV	Leave-one-out cross-validation
MC-GN	Minimal-change glomerulonephritis
MEST-C	Oxford classification of IgA nephropathy
NPV	Negative predictive value
PCR	Urinary protein-to-creatinine ratio
PET	Positron emission tomography
PLA2R	Phospholipase A2 receptor
PPV	Positive predictive value
ROC	Receiver-operating-characteristic
SD	Standard deviation
SGLT2-i	Sodium–glucose co-transporter-2 inhibitor
SLE-LN	Systemic lupus erythematosus with lupus nephritis
SUV	Standardized uptake value
TMA	Thrombotic microangiopathy
VUS	Variant of uncertain significance
WES	Whole-exome sequencing
