# Supplementary material for: Diagnostic Performance of [68Ga]Ga-FAPI PET/CT for Differentiating Histologically Confirmed Kidney Fibrosis Severity in Advanced Chronic Kidney Disease
Source: Diagnostics (Basel). 2026 Aug 25;16(17):2706. doi: 10.3390/diagnostics16172706 (PMC13565492; doi:10.3390/diagnostics16172706)
Supplement: Supplementary file 1 [file diagnostics-16-02706-s001.zip › diagnostics-4480704-Supplemental material_FAPiPETCT_final.pdf]

Supplemental material

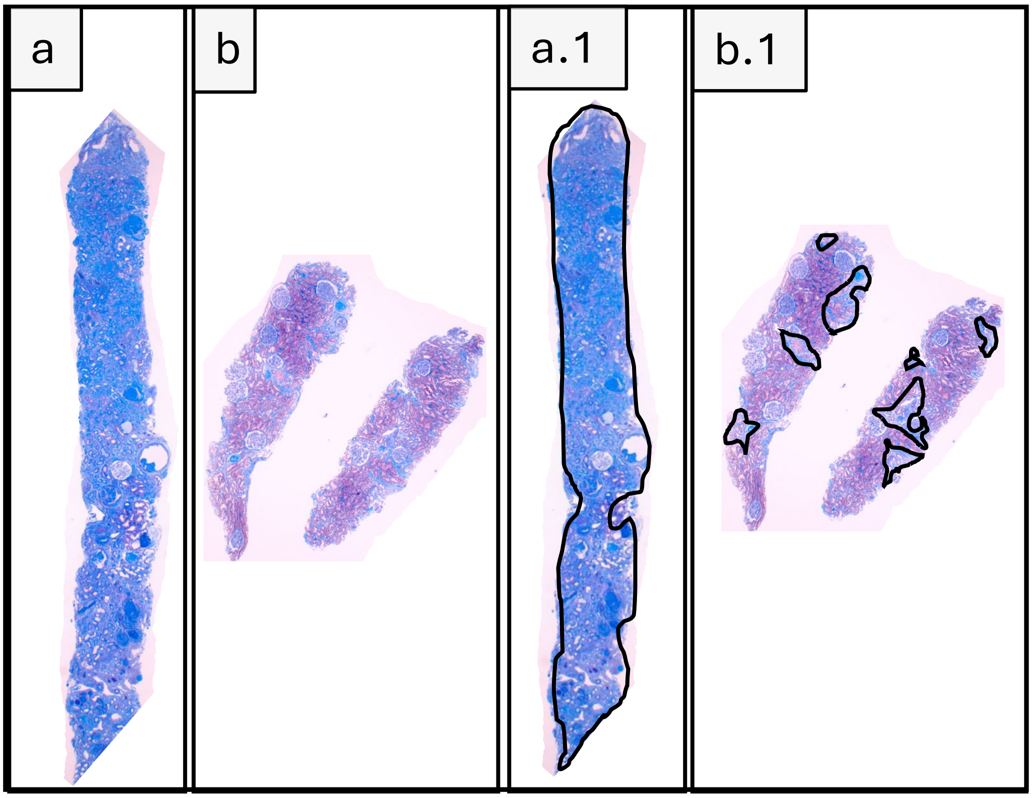

Supplemental Figure S1 Acid fuchsin orange-G Staining of two examples of varying amount of interstitial fibrosis, illustrating scoring of total area of fibrosis by eyeballing. Panel a) shows a biopsy core with 100% high grade fibrosis, with encircled area in Panel a.1, panel b) shows two biopsy cores with multiple areas of mild and moderate fibrosis, in total about 30%, encirceled in b.1.

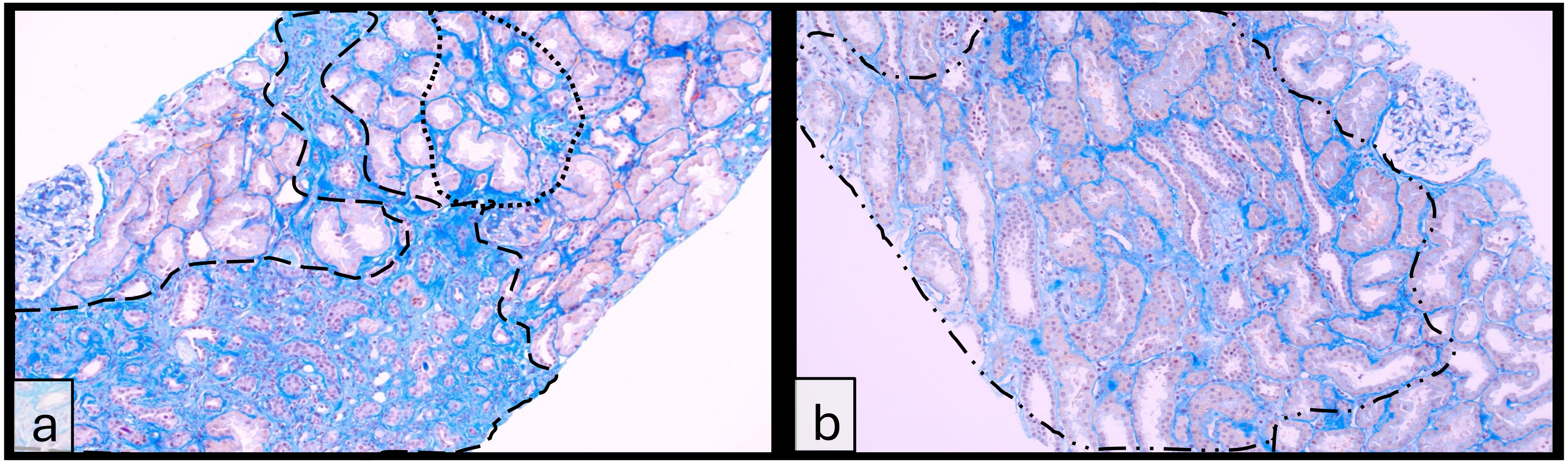

Supplemental Figure S2 Acid fuchsin orange-G Staining of two examples of varying amount of interstitial fibrosis illustrating intensity scoring. Panel a) shows areas of severe (dashed line) and moderate (dotted line) fibrosis, scored as intensity grade III as most if the fibrosis present is of severe grade. Panel b) shows an area of mild interstitial fibrosis, graded as intensity grade I.

The H-Score for these panels would be composed as :
in a) 40% grade III, 10% grade II, 0% grade I and 50% grade0 ; 40x3+10x2+50x0 = final H-score: 140
in b) 0% grade III, 0% grade II, 80% grade I, 20% grade 0 ; 0x3+0x2+80x1+20x0 = final H-score: 80

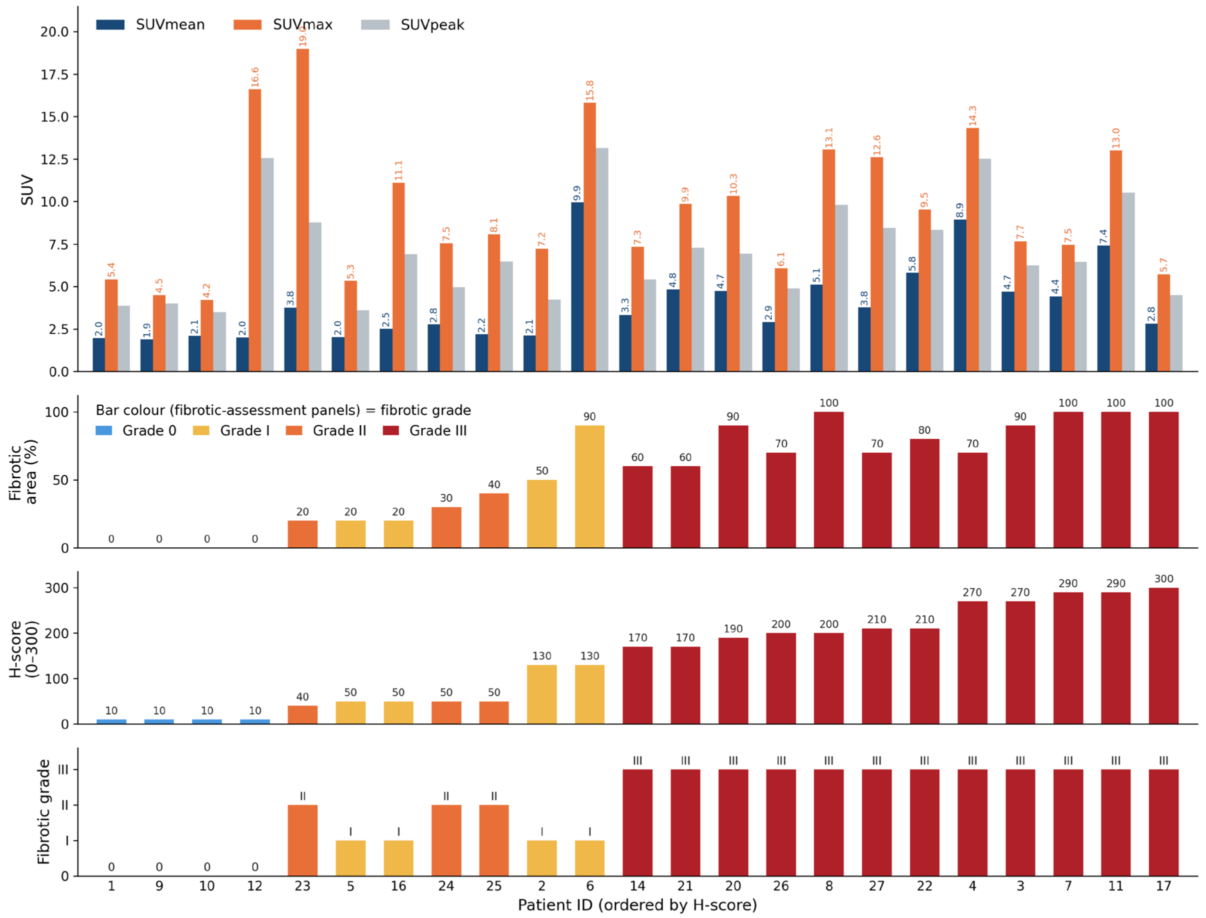

Supplemental Figure S3 SUVmean, SUVmax, SUVpeak and Histologic assessment of included patients SUVmean increases with the degree of fibrosis found in the kidney biopsy, evaluated by fibrotic area, H-score and fibrotic grade. Shown are the values of the biopsied kidney and patients are ordered on behalf of their H-score.

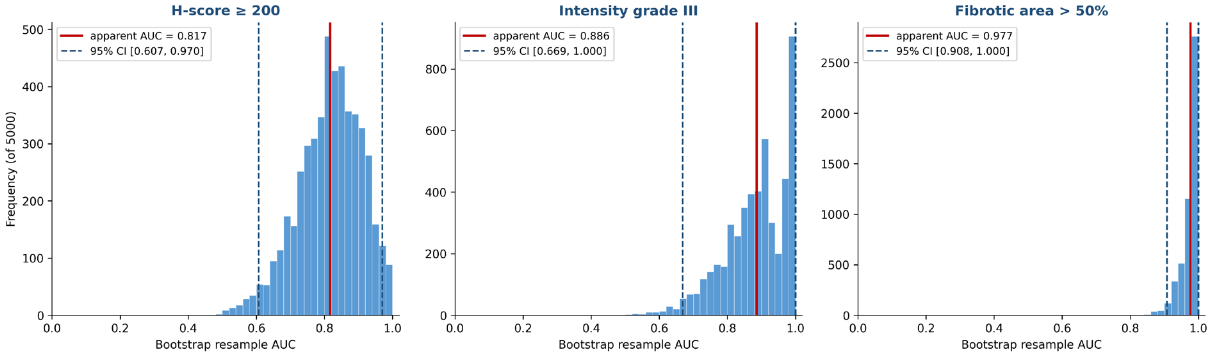

Supplemental Figure S4 Bootstrap distribution of the AUC of renal parenchymal SUVmean for the identification of advanced interstitial fibrosis. Bootstrap resampling confirms the diagnostic performance of SUVmean. Depicted is the ROC curve (AUC) with its bootstrap percentile 95% confidence interval. AUC: area under the curve; CI:confidence interval; SUVmean: mean standardized uptake value

ID	Indication for kidney biopsy	Biopsy type	Days since Bx	Serum creatinine at biopsy (mg/dL)	eGFR at biopsy (mL/min/1.73 m²)	Serum creatinine at imaging (mg/dL)	eGFR at imaging (mL/min/1.73 m²)	Disease-specific therapy between biopsy and PET/CT
1	Proteinuria	Initial biopsy	52	0.84	86.2	0.71	105.5	None
2	Decreased renal function with proteinuria	Initial biopsy	68	1.37	56.3	1.30	60.0	None
3	Decreased renal function and evaluation of SLE activity	Re-biopsy	88	4.60	11.4	4.20	12.7	Unchanged: Mycophenolate sodium 360 mg OD and hydroxychloroquine 200 mg OD continued. History of steroids (Prednisone)
4	Assessment of continuation/switch of immunosuppression	Re-biopsy	80	5.18	10.0	10.30	4.4	Mycophenolate mofetil 1 g BID discontinued three weeks after biopsy (prior MMF and steroids)
5	Proteinuria and hematuria	Initial biopsy	69	n.a.	n.a.	0.55	114.4	None
6	Assessment of continuation/switch of immunosuppression	Re-biopsy	148	9.54	5.9	11.40	4.7	No change; Rituximab 1 g 9/2025 with no further administration after biopsy (history: rituximab 2 × 1 g 2022; MMF until 1/2022, stopped due to anemia)
7	Decreased renal function with proteinuria and hematuria	Initial biopsy	129	5.02	10.7	4.05	13.9	None
8	Decreased renal function	Initial biopsy	194	3.09	21.4	2.80	24.1	None
9	Proteinuria	Initial biopsy	410	0.45	134.3	0.61	124.8	Steroids (Prednisone; non-response after 3 months), then tacrolimus for 3 months (target 4–7 ng/mL) with remission; relapse after tacrolimus discontinuation 11/2025, remission again within 2 weeks; imaging 1/2026 in remission

10	Decreased renal function and hematuria	Initial biopsy	46	1.34	57.9	0.63	129.4	Steroids (Prednisone)
11	Assessment of continuation of immunosuppression and decreased renal function	Re-biopsy	140	9.41	6.9	9.60	6.8	None (History: steroids for 16 weeks from 2024; rituximab 2 × 1 g 6/2025 and 9/2025; no additional immunosuppression between biopsy and imaging)
12	Proteinuria and hematuria	Initial biopsy	82	1.05	94.3	0.86	115.1	None
14	Proteinuria and hematuria	Initial biopsy	60	1.40	56.5	1.30	61.7	None
16	Decreased renal function	Initial biopsy	214	1.40	59.0	1.30	64.5	None
17	Decreased renal function	Initial biopsy	204	3.34	19.9	2.75	25.1	None
20	Decreased renal function and proteinuria	Initial biopsy	172	2.56	25.4	3.50	17.5	None
21	Decreased renal function and laboratory features of TMA	Re-biopsy	60	3.09	24.6	2.59	30.4	Complement inhibition (eculizumab 4 × 900 mg, then 1 × 1200 mg)
22	Decreased renal function and proteinuria	Initial biopsy	34	2.76	28.5	2.80	28.0	None
23	Proteinuria and hematuria	Initial biopsy	34	1.10	72.2	1.15	68.5	None
24	Decreased renal function with proteinuria and hematuria	Initial biopsy	74	n.a.	n.a.	1.48	57.3	None
25	Proteinuria and hematuria	Initial biopsy	277	1.05	94.3	1.25	76.5	None
26	Decreased renal function with hematuria	Initial biopsy	134	1.57	55.7	1.77	48.3	None (Mycophenolate started after imaging)

27	Decreased renal function with proteinuria and hematuria	Initial biopsy	83	2.40	30.7	2.20	34.1	SGLT2 inhibitor and budesonide started one month after biopsy

Supplementary Table S1. Biopsy indication, clinical course of renal function and disease-specific therapy between kidney biopsy and [^{68}Ga]Ga-FAPI PET/CT. *All kidney biopsies were performed for routine clinical indications; 18 of 23 were initial diagnostic biopsies and 5 of 23 were clinically indicated re-biopsies (patients 3, 4, 6, 11 and 21). Serum creatinine and eGFR did not change systematically between the two time points (Wilcoxon $p = 0.59$ and $p = 0.47$, respectively; Spearman correlation between time points $\rho \geq 0.95$; $n = 21$ with paired values), and the magnitude of the individual eGFR change was unrelated to the length of the interval ($\rho = -0.003$, $p = 0.99$). BID: twice daily; OD: once daily; eGFR, estimated glomerular filtration rate; MMF, mycophenolate mofetil; n.a., not available; od, once daily; SGLT2, sodium–glucose cotransporter 2; SLE, systemic lupus erythematosus; TMA, thrombotic microangiopathy; n.a: serum creatinine at biopsy not available (patients 5 and 24 due to external referral and no documentation of external lab value).*

ID	Vol L (ml)	SUVmin L	SUVmax L	SUVmean L	SD L	Peak L	Vol R (ml)	SUVmin R	SUVmax R	SUVmean R	SD R	Peak R	Bx side	Fibr. area (% Bx)	Inten-sity	H-score	0%	+%	++%	+++%
**1**	76.17	0.9	5.43	1.97	0.51	3.88	76.81	0.64	6.47	1.68	0.37	4.48	Left	0	0	10	90	10	0	0
**2**	40.95	0.77	6.51	2.01	0.69	4.1	60.5	0.67	7.23	2.11	0.82	4.23	Right	50	I	130	10	70	0	20
**3**	70.02	1.59	7.66	4.7	1.01	6.25	91.13	1.7	8.13	4.84	1	6.72	Left	90	III	270	10	0	0	90
**4**	147.81	1.52	14.33	8.94	2.5	12.52	149.72	1.16	13.82	8.47	2.53	11.58	Left	70	III	270	0	10	10	80
**5**	108.79	0.69	5.33	2.02	0.43	3.6	98.47	0.59	5.19	1.92	0.55	3.79	Left	20	I	50	70	10	20	0
**6**	97.3	1.86	15.82	9.95	2.49	13.15	110.7	1.45	15.78	9.59	2.5	12.44	Left	90	I	130	0	80	10	10
**7**	38.92	0.9	8.36	4.88	1.45	7.02	42.91	1.12	7.45	4.43	1.3	6.46	Right	100	III	290	0	0	10	90
**8**	142.7	0.64	13.06	5.12	1.62	9.81	n.a.	n.a.	n.a.	n.a.	n.a.	n.a.	Left	100	III	200	0	30	40	30
**9**	140.36	0.9	4.5	1.9	1.35	4	130.69	0.8	3.6	1.8	1.44	3.6	Left	0	0	10	90	10	0	0
**10**	127.28	1.2	4.2	2.1	0.65	3.5	113.5	1.1	4.1	2.3	0.91	4.1	Left	0	0	10	90	10	0	0
**11**	53.1	2.02	13.01	7.4	1.99	10.52	34.8	0.7	10.4	2.83	1.89	7.91	Left	100	III	290	0	0	10	90
**12**	142.74	0.68	16.6	2	2.11	12.54	136.9	0.43	29.1	2	2.37	16.86	Left	0	0	10	90	10	0	0
**14**	128.65	0.91	7.34	3.34	0.91	5.42	89.09	0.53	9.25	3.24	1.34	7.35	Left	60	III	170	40	0	10	50
**16**	87.16	0.25	13.24	3.69	1.74	7.74	162.23	0.3	11.12	2.52	1.06	6.89	Right	20	I	50	70	10	20	0
**17**	60.61	0.93	5.72	2.83	0.91	4.49	152.7	0.92	4.45	2.19	0.4	3.32	Left	100	III	300	0	0	0	100
**20**	214.9	0.75	10.33	4.74	1.38	6.94	149.28	0.56	9.23	4.57	1.34	6.59	Left	90	III	190	10	0	80	10
**21**	120.42	0.85	9.86	4.82	1.36	7.27	111.97	0.88	10.52	4.88	1.34	8.2	Left	60	III	170	40	0	10	50
**22**	157.55	0.87	9.54	5.81	1.66	8.34	148.56	1.04	9.88	5.71	1.88	8.36	Left	80	III	210	20	10	10	60
**23**	180.37	0.57	18.99	3.76	1.24	8.76	179.23	0.64	18.26	3.64	1.33	12.06	Left	20	II	40	80	0	20	0
**24**	143.44	0.63	7.55	2.77	0.79	4.97	132.73	0.57	5.94	2.66	0.86	4.81	Left	30	II	50	70	10	20	0
**25**	259.88	0.55	8.07	2.18	0.62	6.48	224.67	0.51	10.29	2.2	0.69	6.85	Left	40	II	50	70	10	20	0
**26**	144.16	0.89	6.07	2.91	0.84	4.89	140.44	0.64	6.81	2.68	0.89	5.08	Left	70	III	200	30	0	10	60
**27**	202.88	0.3	12.6	3.77	1.67	8.45	179.22	0.45	9.08	3.93	1.49	6.56	Left	70	III	210	30	0	0	70

Supplemental Table S2 Renal [68Ga]Ga-FAPI PET/CT uptake parameters (both kidneys) and histological fibrosis findings of the biopsied kidney SUV parameters are given separately for the left (L) and right (R) kidney; histology refers to the biopsied kidney. SUV: Standardized uptake value (min, max, mean and peak); SD: Standard deviation of the SUV; Vol: Volume; L: Left kidney; R: Right kidney; Bx: Biopsy; Intensity: Interstitial fibrosis intensity grade (0, I, II, III); H-score: Histological fibrosis score (sum of the percentage of stained tissue multiplied by its staining intensity; range 0–300); n.a.: Not available, patient 8: Solitary kidney ;. Fibr. area (% Bx): Morphological fibrotic area as a percentage of the biopsy. 0%, +%, ++%, +++% give the percentage of biopsy tissue with no (0), weak (+), moderate (++) or strong (+++) staining intensity and sum to 100% per patient; this staining-intensity distribution is a separate measure from the morphological fibrotic area

ID	Furosemide (mg/24h)	Spironolactone (mg)	Eplerenone (mg)	HCT (mg)	Xipamide (mg)	SGLT2-i	ACE/ARB agent
**1**	–	–	–	–	–	–	–
**2**	–	–	–	–	–	–	Ramipril 10mg
**3**	80	–	–	–	–	–	Lisinopril 40mg
**4**	375	–	–	–	–	–	Candesartan 8mg
**5**	–	–	–	–	–	–	–
**6**	500	–	–	–	–	–	–
**7**	120	–	–	–	–	–	Ramipril 10mg
**8**	–	–	–	–	–	Empagliflozin	–
**9**	–	–	–	–	–	–	–
**10**	–	–	–	–	–	–	–
**11**	500	–	–	–	–	–	Candesartan 8mg
**12**	–	–	–	–	–	Dapagliflozin	Candesartan
**14**	–	–	–	12.5	–	Dapagliflozin	Candesartan 32mg
**16**	–	–	–	–	–	Dapagliflozin	Candesartan 16mg
**17**	160	–	50	–	5	Dapagliflozin	–
**20**	160	–	–	–	–	Dapagliflozin	Candesartan
**21**	–	50	–	–	–	Dapagliflozin	Sacubitril,Valsartan 49/51mg BID
**22**	–	–	–	–	–	–	Candesartan 32mg
**23**	–	–	–	–	–	–	Enalapril 40mg
**24**	–	–	–	–	–	Dapagliflozin	Candesartan 16mg
**25**	–	50	–	–	–	Dapagliflozin	Telmisartan 120mg
**26**	–	–	–	12.5	–	Dapagliflozin	Irbesartan 300mg
**27**	–	–	–	–	–	Dapagliflozin	Ramipril 5mg

Supplemental Table S3 Antihypertensive, diuretic and SGLT2-inhibitor medication at the time of [68Ga]Ga-FAPI PET/CT. Values are daily doses in mg; „–„ indicates the drug was not prescribed. HCT: Hydrochlorothiazide; SGLT2-i: Sodium–glucose co-transporter-2 inhibitor; ACE: Angiotensin-converting-enzyme inhibitor; ARB: Angiotensin-receptor blocker; BID: twice daily.

Fibrosis measure	Agreement statistic	Estimate (95% CI)	Descriptive agreement
H-score (0–300)	ICC, absolute agreement	0.962 (0.924–0.981)	Bland–Altman bias −1.3, 95% LoA −63.7 to 61.0
Fibrotic area (%)	ICC, absolute agreement	0.961 (0.911–0.986)	Bland–Altman bias +2.6%, 95% LoA −18.9 to 24.1
Intensity grade (0–III)	Weighted kappa (quadratic)	0.912 (0.811–0.972)	Exact agreement 17/23 (74%); within one grade 23/23 (100%)
Advanced fibrosis (intensity grade III vs 0–II)	Cohen's kappa	0.827 (0.572–1.000)	Concordant 21/23 (91%); discordant patients 20 and 26 (grade II vs III)
Advanced fibrosis (H-score ≥ 200)	Cohen's kappa	0.732 (0.401–1.000)	Concordant 20/23 (87%); discordant patients 14, 21 and 26

Supplementary Table S4 Interobserver agreement of the histological fibrosis scoring Biopsies were scored independently by two renal pathologists. Intra-class correlation coefficients (ICC; two-way model, single measure, absolute agreement) were used for the two continuous measures. The corresponding consistency ICCs were 0.960 for the H-score and 0.961 for the fibrotic area. Agreement on the ordinal intensity grade was quantified by quadratic weighted kappa (linear weighted kappa: 0.801, 95% CI 0.627–0.934) and agreement on the two pre-specified dichotomous definitions of advanced fibrosis by Cohen's kappa. All 95% confidence intervals are percentile bootstrap intervals based on 10,000 subject-level resamples with a fixed random seed. CI, confidence interval; ICC, intra-class correlation coefficient; LoA, limits of agreement

Variable	Grades 0–I (n = 8)	Grade II (n = 3)	Grade III (n = 12)	p
Age, years — median [IQR]	41.0 [26.3–49.3]	50.0 [43.0–60.0]	44.0 [42.8–63.0]	0.30
Male sex — n	3/8	3/3	9/12	0.11
Serum creatinine, mg/dL — median [IQR]	0.78 [0.62–1.30]	1.25 [1.20–1.36]	2.80 [2.49–4.09]	0.006
eGFR, mL/min/1.73 m² — median [IQR]	109.5 [63.0–117.5]	68.0 [62.5–72.5]	24.5 [13.8–31.0]	0.005
PCR, mg/g — median [IQR]	404 [203–1223]	1300 [1250–1550]	1242 [680–3776]	0.32
ACR, mg/g — median [IQR]	219 [70–943]	1000 [1000–1250]	952 [387–2571]	0.32
Hematuria — n	3/8	3/3	3/12	0.070
SGLT2 inhibitor — n	2/8	2/3	7/12	0.36
ACE inhibitor / ARB — n	3/8	3/3	10/12	0.080
Loop diuretic — n	1/8	0/3	6/12	0.11
Biopsy-to-PET interval, days — median [IQR]	75.5 [64.0–164.5]	74.0 [54.0–175.5]	108.5 [75.0–148.0]	0.93

Supplemental Table S5 Baseline clinical variables according to fibrosis intensity grade. Serum creatinine and eGFR differed significantly across the three groups, whereas age, sex, proteinuria, albuminuria, hematuria, medication use and the biopsy-to-imaging interval did not differ significantly.

CKD stage	n	SUVmean, median [IQR]
G1–G2 (eGFR ≥ 60)	10	2.10 [2.00–2.44]
G3a–G3b (eGFR 30–59)	4	3.34 [2.88–4.03]
G4–G5 (eGFR < 30)	9	5.12 [4.70–7.40]

Supplemental Table S6 Renal parenchymal SUVmean stratified by CKD stage. CKD stages were defined by the eGFR at the time of imaging according to KDIGO categories (G1–G2: ≥ 60; G3a–G3b: 30–59; G4–G5: < 30 mL/min/1.73 m²). Values are median [IQR: interquartile range].CKD: Chronic kidney disease

Endpoint	Mean	Median	SD	Min	2.5th	97.5th	Max	% at 1.000
**H-score ≥ 200**	0.815	0.823	0.093	0.230	0.607	0.970	1.000	0.9%
**Intensity grade III**	0.887	0.897	0.091	0.454	0.669	1.000	1.000	13.3%
**Fibrotic area > 50%**	0.977	0.985	0.029	0.714	0.908	1.000	1.000	38.5%

Supplemental Table S7 Bootstrap distribution statistics (B = 5000) confirms the diagnostic performance of SUVmean. AUC: area under the curve; SD:standard deviation; SUV: standardized uptake value; SUVmean: mean standardized uptake value
